# A Novel Quick Release Mechanism for Ankle Foot Orthosis Struts

**DOI:** 10.33137/cpoj.v5i2.38802

**Published:** 2022-12-18

**Authors:** W Li, N Baddour, E.D Lemaire

**Affiliations:** 1 Department of Mechanical Engineering, University of Ottawa, Ottawa, Canada.; 2 Department of Medicine, Faculty of Medicine, University of Ottawa, Ottawa, Canada.; 3 Centre for Rehabilitation Research and Development, Ottawa Hospital Research Institute, Ottawa, Canada.

**Keywords:** Ankle Foot Orthosis, Dynamic Gait Rehabilitation, Multi-Stiffness Ankle Foot Orthosis, Quick Release, Orthosis

## Abstract

**BACKGROUND:**

A posterior dynamic element ankle-foot orthosis (PDEAFO) uses a stiff carbon fibre strut to store and release energy during various mobility tasks, with the strut securely attached to the foot and shank-cuff sections. A design that allows the user to swap struts for specific activities could improve mobility by varying PDEAFO stiffness, but current approaches where bolts securely connect the strut to the orthosis make quick strut swapping time-consuming and impractical.

**OBJECTIVES:**

Design a novel quick release AFO (QRAFO) that can enable daily living strut-swapping and thereby enable better ankle biomechanics for the person’s chosen activity.

**METHODOLOGY:**

The novel QRAFO enables device stiffness changes through a quick release mechanism that includes a quick-release key, weight-bearing pin, receptacle anchor, and immobilization pin. A prototype was modelled and simulated with SolidWorks. Mechanical tests were performed with an Instron 4482 machine to evaluate quick release mechanism strength with running and 20° slope downhill walking loads. Quick release efficiency was then evaluated via two quick release functional tests, with four participants wearing a 3D printed QRAFO.

**FINDINGS:**

Simulated stress on the weight bearing pin, anchor, and surrounding carbon fibre structure under running and downhill walking loads did not exceed the yielding stress. Mechanical tests verified the simulation results. Four participants successfully swapped the strut within 25.01 ± 3.66 seconds, outperforming the 60.48 ± 10.88 seconds result for the hand-tightened bolted strut. A learning evaluation with one participant showed that, after approximately 30 swapping iterations, swap time was consistently below 10 seconds.

**CONCLUSION:**

The quick release mechanism accommodated running and slope walking loads, and allowed easy and fast strut removal and attachment, greatly reducing strut swap time compared to screw-anchor connections. Overall, the novel quick release AFO improved strut-swapping time without sacrificing device strength, thereby enabling people to use the most appropriate AFO stiffness for their current activity and hence improve mobility and quality of life.

## INTRODUCTION

An ankle foot orthosis (AFO) improves mobility by diminishing foot drop during swing phase and providing gait control during stance.^1^ A recent advancement in AFO design used a posterior strut to store and return energy during movement. An appropriate posterior strut can be selected to accommodate the user’s need for AFO stiffness based on their weight and activity level.^2,3^ However, further functional improvements could be achieved if the person could have different AFO stiffness depending on their chosen activity. For example, less stiffness for driving a car, medium stiffness for walking, high stiffness for high-active movements (running, downhill walking etc.).

The Intrepid Dynamic Exoskeletal Orthosis (IDEO) is an energy storing device that supports and protects users following lower extremity limb salvage procedures.^4^ This AFO was crafted with three carbon fiber components: ground reaction cuff for circumferential support providing off-loading to alleviate ankle pain, posterior strut that deforms for energy storage and return, and footplate. The IDEO modular design allows strut changes as motion ability changes and can be easier to don and doff.^4,5^ The Posterior Dynamic Element AFO (PDEAFO), developed by Fabtech Systems (Everett, WA, USA), is a commercial AFO fabricated entirely from carbon fiber. Similar to IDEO, the PDE AFO consists of a stiff strut that stores energy during weight loading and stance, and returns the energy during late stance. The strut attaches to the AFO shank and sole through bolts, secured with Locktite. An anchor system is integrated by laminating a pre-threaded metal plate within the carbon fibre matrix, thereby facilitating strut adjustment while customizing. The strut stiffness and dimensions can be selected to match the user’s activity level.

A modularized posterior strut AFO design provides possibilities for strut swapping, thereby swapping AFO stiffness. However, bolt connections between the strut and AFO prevent effective strut changing during the day (i.e., requires tools, more time, etc.). A quick release connection between the strut and AFO could be an alternative that enables fast strut-swapping to change stiffness for different activities.

The purpose of this research was to develop a novel quick release AFO (QRAFO) that provides safe and secure energy storage and return, but also allows the QRAFO user to swap struts within 30 seconds, to provide appropriate stiffness for their current activity. Upon successful simulation, mechanical, and functional tests, the QRAFO could be used in daily living to enhance mobility and thereby improve quality of life.

### Quick release mechanism design

**Figure 1:**
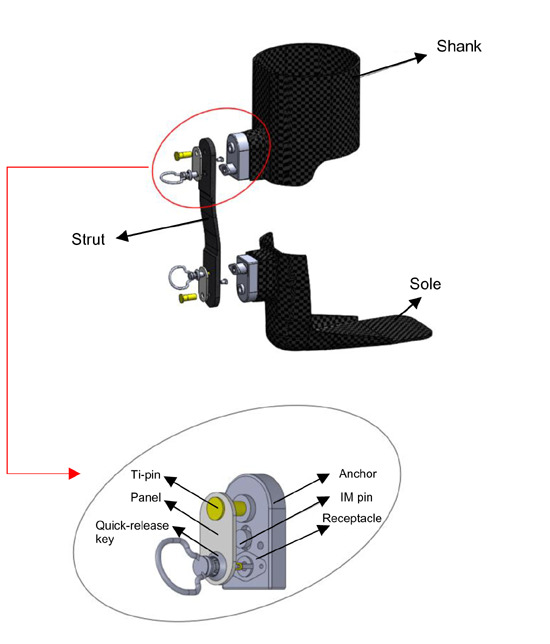
Quick release AFO with quick release mechanism

The new quick release mechanism^6^ (**Figure 1**) consists of five components: quick release key, weight bearing pin, receptacle, anchor, and immobilization pin. The quick release key is affixed on the strut and the anchor is affixed on the receptacle. A panel between the anchor and quick release key fits the gap between the strut and orthosis when installing thinner struts. The anchor is moulded into the AFO. Pushing and twisting the quick release key allows the user to pull the strut out of the anchor. A titanium alloy weight bearing pin (Ti-pin) bore most of user’s weight during movement. To prevent strut rotation along the weight bearing pin, an immobilization pin (IM pin) was included between the quick release key and weight bearing pin.

The weight bearing pin was designed to bear all transverse forces on the quick release mechanism (QRM) during movement. Titanium alloy Ti-6Al-4V was selected due to its high yield and ultimate strength. To construct a lightweight device, aluminium 6061 was selected for the anchor. Quick release key and receptacle were also made of aluminium 6061 due to its light weight and appropriate strength. The total QRM weight was 30 g.

## METHODOLOGY

Three analyses were performed to assess QRAFO strength and functionality. FEA simulation was performed on the QRM, including yielding analysis and safety factor analysis under walking load (fatigue load), running load (intense load), and downhill walking load (bending load). Mechanical testing was performed on the QRM to analyse the stress-displacement curve and material deformation. QRM functional testing compared QRM strut swap efficiency to the PDEAFO screw-anchor mechanism.

### Strength analysis

#### Finite Element Analysis

**Figure 2:**
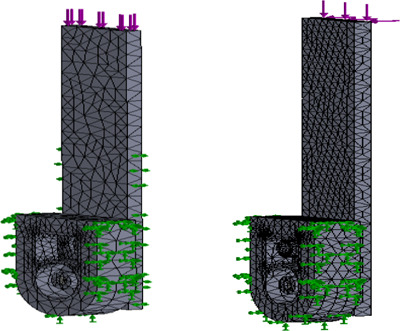
Load and fixture conditions on meshed QRM with two loading types: shearing caused by either walking or running (Left); bending caused by landing on uneven ground, representing downhill walking load (Right).

A QRAFO for daily use must not fail during occasional intense activities and long periods of walking. SolidWorks 2019 (Dassault Systèmes, Vélizy-Villacoublay, France) was used to perform finite elements analysis (FEA) to simulate the load exerted on the quick release mechanism under three scenarios: level walking, running, and downhill walking (**Figure 2**). The designed device capacity was based on a 120 kg user.

Only shear loads were considered for daily walking and running loads, while bending was included in downhill walking. The 95^th^ percentile Canadian male weighs 113.5 kg.^7^ Considering that users may carry personal belongings, a QRAFO should withstand daily use by a 1200 N person. Peak ground reaction forces for testing were bodyweight for walking,^8^ 3 times bodyweight for running,^9^ and 1.2 times bodyweight for downhill walking.^8^ Considering the AFO cuff off-weighting function (i.e., supporting body weight for some AFO applications), the vertical force applied on the QRM was 80% of the peak ground reaction forces.^10^ The QRM was modelled with virtual jigs (strut to apply load, fixed quick release male components, and shell to fix quick release female components, **Figure 2**). Shearing and bending loads were applied to the top of the strut (250 mm long). The shell was globally fixed.

Since AFO devices are suggested to last three years^11^ with 10,000 walking steps per day as a common goal for adults,^12^ the QRM should last 10^7^ regular walking cycles.

### Mechanical Tests

**Figure 3:**
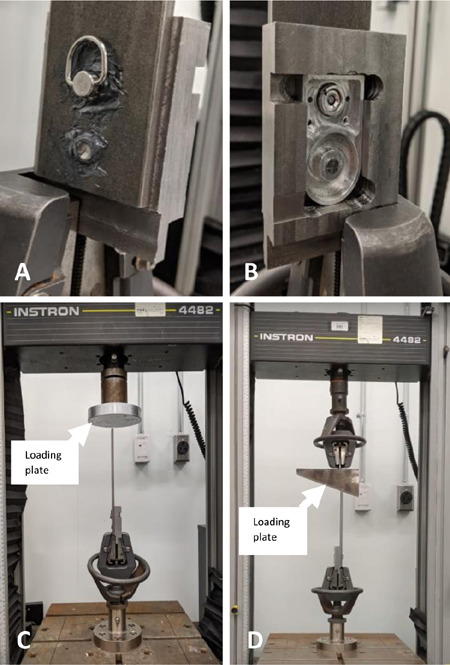
Anchor fixed to vertical loading strut (i.e., represents shank connection) (A); quick release key and weight bearing pin fixed to clamp (i.e., represents foot-ankle unit connection) (B); Testing setup for running load (C); downhill walking load with angled loading plate (D).

Mechanical tests were performed with running and downhill walking loads using an electromechanical testing machine (4482, InstronR, Norwood, MA) with a 10 kN static load cell (10 N resolution, ISO-376, InstonR, Norwood, MA). 2880 N maximum force was applied at a constant speed of 1 mm/min for the running load and 1080 N maximum vertical force at the same speed for a 20-degree downhill walking load. A special triangular fixture with a surface angle of 20 degrees was machined to apply a moment to the quick release mechanism (**Figure 3**). The load cell initial position was manually set to approximately one millimetre from the iron shell. A smartphone was fixed on a tripod to video record the trials. When the force sensed by the load cell reached the maximum load or the displacement reached 10 mm, the load cell terminated action and returned to the origin position. Ten trials were collected and analysed for each test.

The Instron machine recorded data at 10 Hz. After testing, the force-displacement relation was explored by analysing the slope of the curve. Quick release component dimensions were measured by a caliper (Accusize Industrial Tools, AB11-1106) before and after testing to determine if surface damage occurred between the Ti-pin and aluminum anchor.

### Functional Analysis

Two AFOs were 3D printed for the functional analysis. The two AFOs had identical components but different connection mechanisms: one with the quick release mechanism and another with a PDEAFO screw-anchor mechanism. While screws are typically secured using Locktite to ensure that the strut does not loosen, the screws were hand tightened for this test to enable comparison.

Four able bodied participants were recruited (3 males, 1 female). Ethical approval was received from the University of Ottawa Research Ethics Board (File number H-10-19-4767). All participants provided informed consent.

While sitting on a chair, participants donned the AFO with quick release mechanism. After self-finding a comfortable position, the participant removed the strut, waited 2 to 4 seconds, and then reattached the strut. This swap trial was performed 10 times. Then, the participant donned the AFO with the screw anchor mechanism and repeated the swap trial 10 times. All swap trials were recorded with a GoPro camera (San Mateo, California, USA) affixed on a tripod. All participants were asked to adjust their posture and position to provide a clear side view to the camera.

To investigate the learning process for strut swapping, one participant performed the strut swap trial 50 times for each device. The time to complete each strut removal and each strut attaching were extracted from the digital video using MATLAB R2019b (MathWorks, Natick, Massachusetts, USA). Strut removal started when the hand touched any strut component and ended when all strut components were not contacting the AFO. Strut removal with the screw-anchor connection started when the screwdriver touched any strut component and ended when all strut components were not contacting the AFO. Strut attaching with the QRM started when any strut component touched the AFO and ended when hand not contacting the AFO. Strut attaching with the screw-anchor connection started when any strut component touched the AFO and ended when the screwdriver was not touching any strut components.

## RESULTS

### FEA simulation

**Figure 4:**
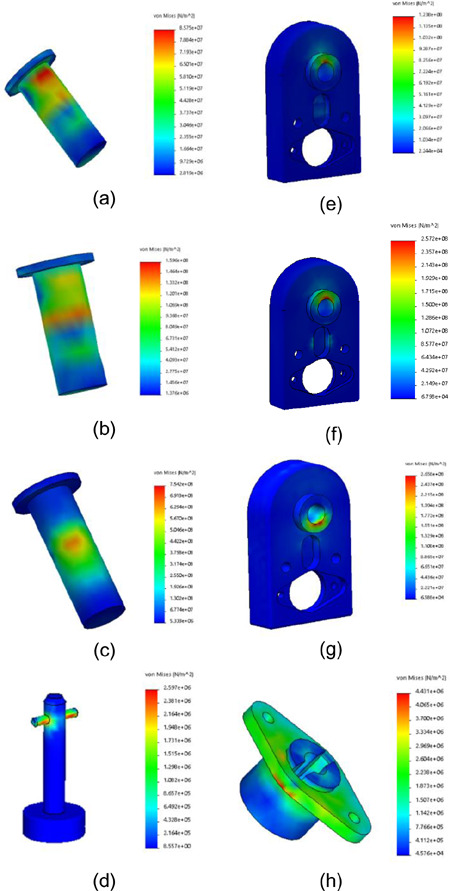
Simulation results for QRM components: stress distributions of weight bearing pin with (a) walking load; (b) running load; (c) downhill walking; (d) stress distribution of quick release key with downhill walking load; (e) stress distributions of anchor with walking load; (f) running load; (g) downhill walking; and (h) stress distribution of receptacle with downhill walking load.

The Modified Goodman equation was used to calculate the safety factor of fatigue given by:


(1) $$\frac{\sigma_{a}}{S_{e}}+\frac{\sigma_{m}}{S_{ut}}=\frac{1}{n}$$

where σ_*a*_ is the amplitude component of stress, σ_*m*_ is the midrange stress component, **S_e_** is the fatigue strength, and **S_ut_** is the ultimate strength and **n** is the safety factor. The fatigue strength of aluminium is 117 MPa and grade 5 titanium is 280 MPa.^10^ **Figure 4** shows the stress distributions over QRM components with the three loads.

By assuming the amplitude and midrange stress are equal (i.e., half the maximum stress) the safety factor to fatigue under walking load was 5.09 for the weight bearing pin and 1.37 for the anchor. Compared with the Ti-pin (880 MPa) and anchor (270 MPa) yielding strengths, yielding safety factors were 5.5 for the Ti-pin and 1.07 for the anchor with running load. Downhill walking load produced more stress on the components, with yielding safety factors of 1.17 for Ti-pin and 1.02 for anchor. The bending force also generated a pulling force on the quick release key and receptacle, with safety factors of 10.38 for the quick release key and 6.14 for the receptacle.

### Mechanical testing results

**Figure 5:**
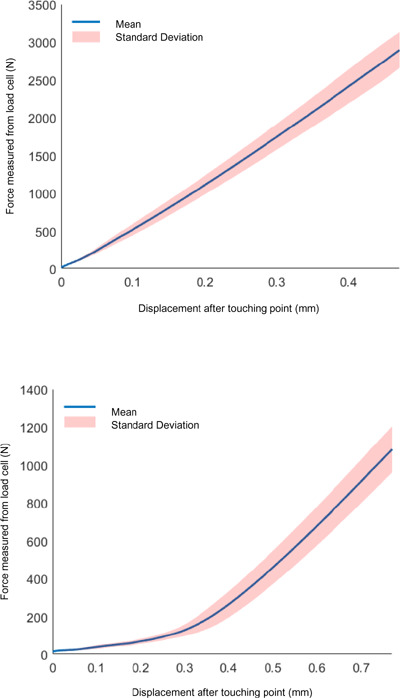
Force-displacement curves from running load test (Top) and downhill walking load test (Bottom).

From the mechanical tests, force-displacement analysis indicated no yielding with running and downhill walking loads, demonstrated by the curve increasing monotonically (**Figure 5**). The QRM materials were in their elastic region when the maximum loads were applied.

Pearson correlation coefficients between force-displacement curves were larger than 0.99. Therefore, both running and downhill walking load tests were repeatable.

**Table 1** shows the mean and standard deviation of the measured dimensions before and after testing. The mean Ti-pin diameter (6.29 mm), Ti-pin length (21.10 mm), and anchor hole diameter (6.38 mm) were within 0.02 mm of their original dimensions. Standard deviations were smaller than 0.02 mm, so measurements along one surface were consistent. Therefore, surfaces were not damaged due to running and downhill walking loads.

**Table 1:** Means and standard deviations (mm) of the original Ti-pin diameter, length, and anchor hole diameter dimensions. Dimensions are before testing, after running load, and after downhill walking load tests.

	Before Testing	Running Load	Downhill Load
Ti-pin diameter (mm)	6.29 (0.01)	6.29 (0.02)	6.28 (0.01)
Ti-pin length (mm)	21.10 (0.01)	21.11 (0.01)	21.09 (0.01)
Anchor hole diameter (mm)	6.37 (0.01)	6.38 (0.01)	6.38 (0.01)

### Quick release efficiency test

**Figure 6:**
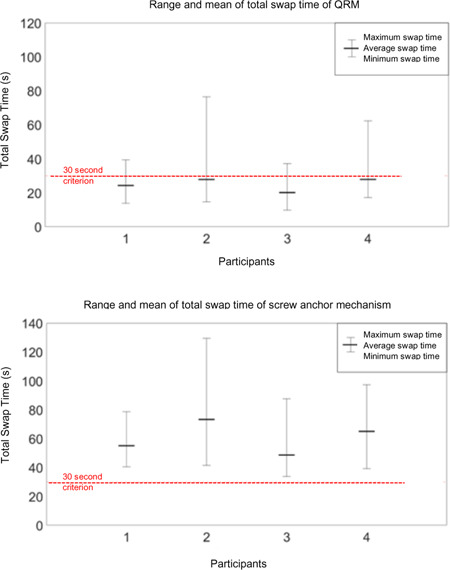
Range and mean of total swap time for QRM and screw anchor connection.

The average swap time across the four participants with QRM was 25.01 ± 3.66 seconds. All participants swapped the strut within 30 seconds, on average (**Figure 6**). The best swap time was 13.83 ± 3.08 seconds and the worst swap time was 53.82 ± 18.90 seconds, among all participants. As a comparison, the average screw anchor mechanism swap time was 60.48 ± 10.88 seconds, 142% longer than QRM swap time. The best swap time was 38.71 ± 3.43 seconds, 180% longer than swap with QRM and the worst swap time was 98.23 ± 22.19 seconds, 83% longer than swapping with QRM. All participants failed to swap screw anchor mechanism struts within 30 seconds (**Figure 6**).

### Strut swap learning

**Figure 7:**
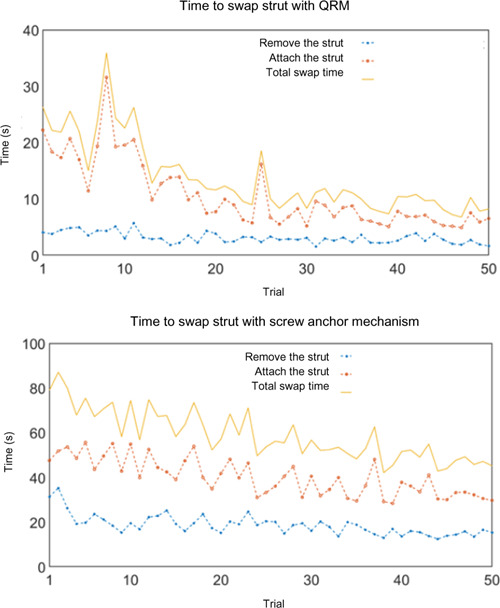
Time to swap strut with QRM and screw-anchor mechanism; including, strut removal time, strut attaching time, and total strut swap time.

For the participant who completed the 50 trial test, the average swap time with the QRM was 13.85 ± 6.52 seconds and with the screw-anchor was 58.41 ± 11.16 seconds. As the participant learned how to best swap the strut, swap time improved from a maximum of 35.95 seconds to 6.81 seconds. **Figure 7** shows the learning effect since strut swapping time decreased over the first 30 trials. The first ten strut swaps averaged 23.97 seconds and the last ten swaps averaged 8.92 seconds. Standard deviation also improved, with a standard deviation of the first ten trials of 5.22 seconds and the last ten trials of 1.43 seconds.

Less time was needed to remove the strut than attach the strut. The average time to remove the strut, over the first ten trials, was 4.27 ± 0.68 seconds and over the last ten trials was 2.68 ± 0.82 seconds (37.2% decrease). QRM swapping time was much less than the 30-second design criteria.

In comparison, the screw-anchor mechanism averaged 58.41 ± 11.16 seconds to swap. A milder learning effect was seen on screw anchor mechanism swapping (**Figure 7**). The average time to swap the strut for the first ten trials was 73.39 ± 7.98 seconds, including a mean attaching time of 50.37 ± 4.71 seconds and a mean removing time of 23.01 ± 6.23 seconds. The average time to swap the strut for the last ten trials was 47.85 seconds, with a mean attaching time of 33.07 seconds and a mean removing time of 14.78 seconds. The time decrease in total swap time between the first ten trials and last ten trials was 34.8%, including a 34.3% decrease in attaching and 35.8% decrease in removing. Standard deviations were also larger than the QRM results. The standard deviation of the first ten trials was 7.98 seconds, and the last ten trials was 3.74 seconds. More time was required to swap struts when using an AFO with the screw-anchor mechanism, and the 30-second swapping criterion was not achieved.

## DISCUSSION

A new quick release mechanism was successfully designed and prototyped to enable a person using a posterior-strut style AFO to quickly swap the strut, enabling different strut stiffnesses that would better relate to the person’s chosen activity. Swap time was below the 30 second target, and with practice can be consistently below 10 seconds. Since the QRM strut can be swapped without tools, this mechanism has a greater potential to be used in daily living than approaches requiring screw drivers or other tools.

Mechanical testing revealed that the QRM could bear running and downhill walking loads for a 120 kg person with no failure from material or connections. Force-displacement curve analysis revealed that QRM materials remained in their elastic region under the maximum target loads. The ten trials showed high repeatability, indicating that the connection was not failing (slipping, dislocating, etc.) under running and downhill walking loads. Titanium did not harm the aluminum anchor’s surface, inferred from low dimension variation between trials.

All QRM component safety factors under walking loading, running load, and downhill walking load were greater than one. The lowest safety factor was for walking on a 20-degree descending hill; however, the mechanical test force-displacement results gave confidence in the design since no plastic deformation occurred under maximum running or downhill walking loads. As well, the Ti-pin did not damage the aluminum anchor surface under large loads since the weight bearing pin and anchor dimensions did not change after each test. Pearson correlation coefficients between trials were close to 1, reflecting high similarity between trials from the same test. While the evidence proved that the QRM can withstand a range of daily activity loads, safety factors close to the yielding margin for highly active users such as athletes would require further testing to verify the QRAFO loading parameters under higher loading conditions.

QRM functional tests revealed that participants can quickly swap struts while sitting. The time was substantially lower than the screw-anchor mechanism swap time. During testing, participants spent more time at the beginning and tended to swap faster after they became accustomed to the swap method and posture. After learning, a user can swap struts in under 10 seconds and with less variability, which outperformed our design criteria. The actual swap time could be much less than we observed in experiments since AFO users would have many more swap instances over the years of AFO use.

Various limitations should be considered for this study. Under extreme cold weather, the high thermal conductivity of aluminum alloy can result in the metal components being cold to the touch when swapping struts. Due to the high tolerance of the Ti-pin and anchor, if small particles such as sand stay inside the anchor more push and pull force could be required while swapping struts and may damage the anchor hole inner surface with prolonged wear. This issue can also occur for muddy roads since mud could stay in the anchor hole, thereby leading to difficulty while swapping struts. These conditions could be mitigated with a proper device cleaning regiment. Though functional tests successfully verified QRM function on a 3D printed AFO, QRM performance with a complete carbon fibre posterior strut AFO was not evaluated. Further testing with a larger sample size is required to confirm QRM performance in daily living environments.

## CONCLUSION

In this research, the quick release strut swapping system of a novel QRAFO was designed and evaluated. The quick-release mechanism allows individuals with dorsiflexor/ plantarflexor weakness to tune their AFO to their daily activities, such as driving, walking, downhill walking, and running. This design was low profile allowing the orthosis to fit beneath regular clothing. The weight added to the strut is minimal, which motivates users to carry extra struts with different stiffness levels for use during the day, or have various stiffness struts in their car, sport bag, or at work. Simulation and mechanical tests demonstrated that the components should withstand running and downhill walking loads. Functional testing showed that people could swap struts quickly, thereby encouraging use in daily living. Future research should evaluate QRAFO use with current posterior strut AFO users.

## DECLARATION OF CONFLICTING INTERESTS

The authors report no conflicts of interest to disclose.

## AUTHORS CONTRIBUTION

**Wentao Li**: Conceptualization, design, methodology, analysis, investigation, writing original draft, data interpretation, ethic certification application.

**Natalie Baddour**: Conceptualization, supervision, methodology, reviewing/revising manuscript, final manuscript approval.

**Edward D. Lemaire**: Conceptualization, supervision, methodology, reviewing/revising manuscript, final manuscript approval.

## SOURCES OF SUPPORT

This project was funded by Natural Sciences and Engineering Research Council of Canada (NSERC).

## ETHICAL APPROVAL

The study was approved by the University of Ottawa Research Ethics Board, University of Ottawa, Canada. Signed informed consent was obtained from each participant before commencing.
